# Parentage and relatedness reconstruction in *Pinus sylvestris* using genotyping-by-sequencing

**DOI:** 10.1038/s41437-020-0302-3

**Published:** 2020-03-02

**Authors:** David Hall, Wei Zhao, Ulfstand Wennström, Bengt Andersson Gull, Xiao-Ru Wang

**Affiliations:** 1 Department of Ecology and Environmental Science, UPSC, Umeå University, Umeå, Sweden; 2 Advanced Innovation Center for Tree Breeding by Molecular Design; College of Biological Sciences and Technology, Beijing Forestry University, Beijing, China; 3 The Forestry Research Institute of Sweden (Skogforsk), Sävar, Sweden

**Keywords:** Forestry, Ecological genetics, Genetic markers

## Abstract

Estimating kinship is fundamental for studies of evolution, conservation, and breeding. Genotyping-by-sequencing (GBS) and other restriction based genotyping methods have become widely applied in these applications in non-model organisms. However, sequencing errors, depth, and reproducibility between library preps could potentially hinder accurate genetic inferences. In this study, we tested different sets of parameters in data filtering, different reference populations and eight estimation methods to obtain a robust procedure for relatedness estimation in Scots pine (*Pinus sylvestris* L.). We used a seed orchard as our study system, where candidate parents are known and pedigree reconstruction can be compared with theoretical expectations. We found that relatedness estimates were lower than expected for all categories of kinship estimated if the proportion of shared SNPs was low. However, estimates reached expected values if loci showing an excess of heterozygotes were removed and genotyping error rates were considered. The genetic variance-covariance matrix (G-matrix) estimation, however, performed poorly in kinship estimation. The reduced relatedness estimates are likely due to false heterozygosity calls. We analyzed the mating structure in the seed orchard and identified a selfing rate of 3% (including crosses between clone mates) and external pollen contamination of 33.6%. Little genetic structure was observed in the sampled Scots pine natural populations, and the degree of inbreeding in the orchard seed crop is comparable to natural stands. We illustrate that under our optimized data processing procedure, relatedness, and genetic composition, including level of pollen contamination within a seed orchard crop, can be established consistently by different estimators.

## Introduction

Quantifying varying levels of kinship and genetic structure are essential for understanding the underlying genetics of complex traits (Hall et al. 2007; Lander and Schork 1994), correctly associating genetic with phenotypic variation (Sillanpää 2011), identifying genetic signals of adaptation (Eckert et al. 2010), reducing inbreeding for conservation and artificial selection regimes in agriculture and forestry breeding (Allendorf et al. 2010; Hayes et al. 2009). Levels of relatedness and genetic structure vary extensively among different organisms and are functions of mating systems, demography, and landscapes.

Mating systems influence many population genetics parameters and especially in plants where propagation, pollination and fertilization biology are more diverse than in animals (Barrett and Harder 2017; Park et al. 2018). Organisms with highly fragmented habitats and limited abilities for long range migration or gene flow have an increased genetic structure (Horton et al. 2012; Tatarenkov et al. 2015), while outcrossing wind pollinated trees with extensive pollen dispersal, such as Scots pine (*Pinus sylvestris)*, show little genetic structuring across their distribution range (Kremer et al. 2012; Lindgren et al. 1995; Pyhäjärvi et al. 2007; Robledo-Arnuncio 2011; Wang et al. 1991). Low genetic differentiation among populations facilitates power to detect associations between genetic and phenotypic variation, but reduces the possibility to identify the origins of genetic samples and alleles based on allele frequencies (Kidd et al. 2014). With low levels of population structure, the origins of the samples used for allele frequency reference become less important than in a highly fragmented population, where in the former large numbers of shared alleles among individuals are more likely to be explained by identity by descent (IBD) than by demographic history (Wang 2011b).

Estimating relatedness among individuals, particularly in non-model organisms, has, until recently, mainly relied on multi-allelic co-dominant markers such as SSRs (simple sequence repeats) (Funda et al. 2016). The use of SNP markers has increased tremendously with high-throughput sequencing methods such as genotyping-by-sequencing (GBS) that can facilitate the production of an extensive SNP-matrix for a large number of individuals at an affordable cost (Pan et al. 2015). GBS has the advantage of genotyping and discovering SNPs simultaneously, removing the need to first identify and optimize a set of markers before analysis of a new species or population (Baird et al. 2008; Davey et al. 2011). Although SSR markers generally provide more information per marker than SNPs, the limited number of useful SSRs for many organisms makes the GBS method superior for assessing diversity and relatedness (Yang et al. 2011). With thousands of SNPs in any one population, the power to infer relatedness accurately is much increased (Attard et al. 2018; Hellmann et al. 2016). GBS relies on sequencing fragments within a subjectively decided range that are replicated between experiments to allow for comparison. There is a potential limit to application of GBS in more advanced genetic studies such as association mapping because which loci are sampled across the genome depend on the size selection step that can reduce repeatability between library preps. The method also suffers from allelic dropout and missing data arising from stochasticity in library sequencing (O’Leary et al. 2018).

The main purpose of studying relatedness in breeding programs has been to assess the degree of coancestry in breeding populations and to monitor the pedigree structure in offspring from crossing experiments. In forest tree breeding, genetically improved seeds for operational forestry are produced in seed orchards that are established with phenotypically selected and tested superior trees (called plus trees). Pollen contribution from surrounding un-improved stands can reduce the expected genetic gain of the orchard crops (termed pollen contamination). The main tools used to estimate pollen contamination to date have been allozymes and SSRs. The pollen contamination estimates based on these tools show large variation even under similar conditions (Di-Giovanni and Kevan 1991; Torimaru et al. 2009). The potential benefit of using GBS is to obtain more reliable results for parentage analysis and to improve breeding efforts with more accurate estimates of shared genetic variation between relatives (Hayes et al. 2009). A large set of SNPs can also facilitate the construction of genomic relatedness matrices (VanRaden 2008), where the expected proportion of shared alleles is an estimate of the additive genetic covariance, and the proportion of loci where individuals share genotypes provides an estimate of the coefficient of dominant genetic covariance (Lynch and Walsh 1998). There have also been studies aiming to estimate the distances of effective pollen dispersal using molecular markers (Funda et al. 2016; Robledo-Arnuncio 2011; Torimaru et al. 2012). Direct observation of this has been difficult because it requires sampling rare mating events. Utilizing seed orchard settings with known candidate parents and their physical map positions for pedigree reconstruction can mitigate some of these difficulties. The proportion of external pollen contributing to a limited gene-pool could provide an indirect estimate of pollen dispersal effectiveness. Over time and across species, the relative gene flow within species can facilitate inferences about their biogeography and ability to track changing climate.

The objectives of this study were to: (1) explore GBS SNP filtering strategies and their reliability in genetic parameter estimations, (2) compare estimation methods that retrieve pedigree information, and (3) apply these methods to compare the genetic diversity of seed orchard crops and natural stands of Scots pine. Seed orchards are good experimental systems for validation of kinship estimates because the candidate parents are known, making it feasible to reconstruct the pedigree of the offspring, for comparison to a theoretical expected mating structure. We expect that establishing an optimized procedure for relatedness estimation utilizing high-throughput sequencing data will be valuable for both breeding and evolutionary applications.

## Material and methods

### Seed orchard

**Fig. 1:**
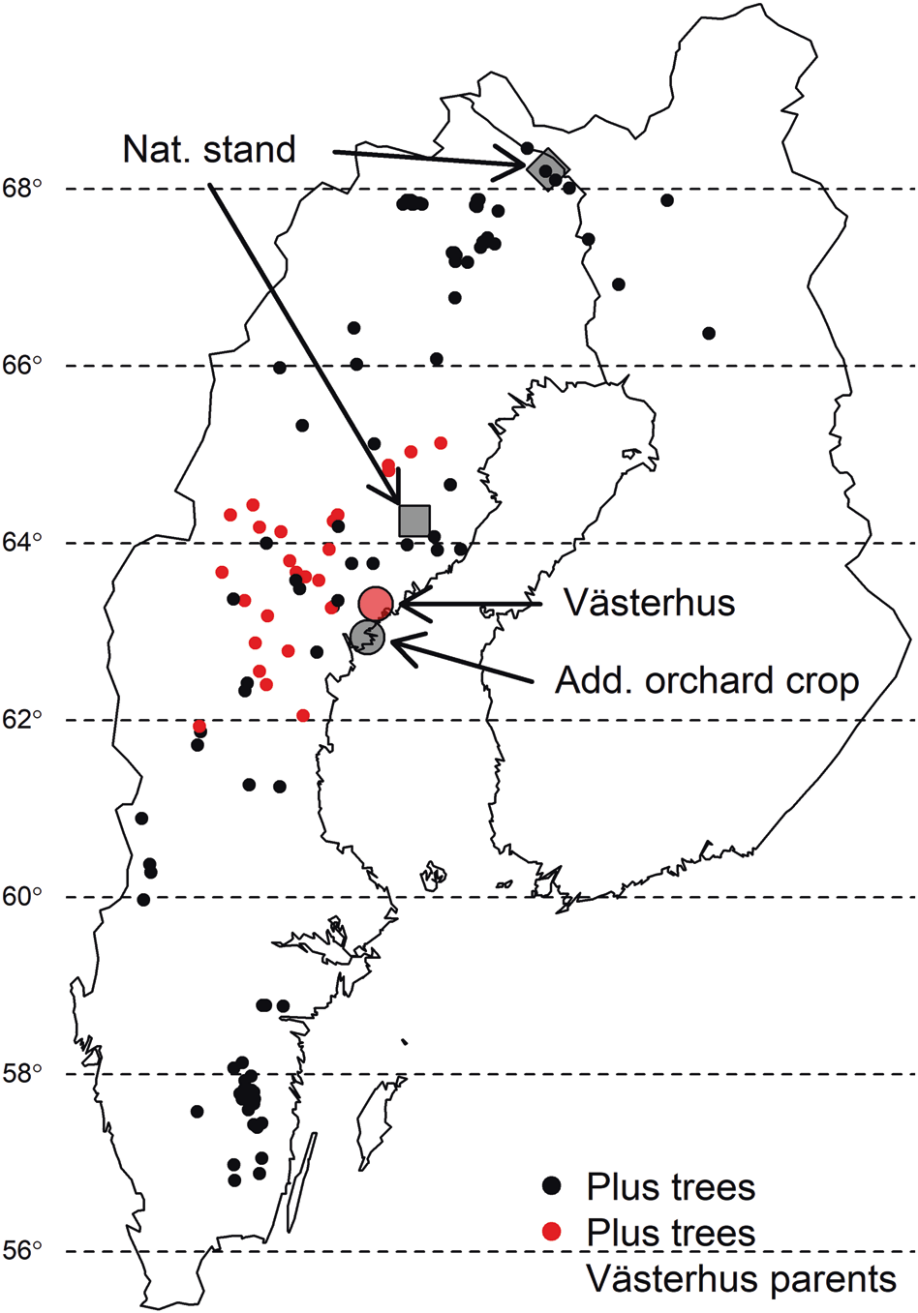
Map of the origin of the samples. Västerhus orchard and parents in red filled circles; additional orchard location in gray (just south of Västerhus), additional plus trees by black filled circles, and natural stands are represented by a black filled diamond (Kuttainen) and square (Lillberget), respectively.

Our study system was the Scots pine seed orchard “Västerhus” (13.7 ha), located in northern Sweden (63 °18′N, 18 °32′E) (Fig. 1). This orchard was established in 1991 using 28 genotypes (parents) following the linear deployment strategy (Lindgren and Matheson 1986), where the number of ramets per genotype is proportional to their breeding values. During the last inventory of the orchard (2011) it contained 3883 ramets with 35 to 332 ramets per genotype, 34 ramets with lost tags, and 67 trees classified as overgrown rootstocks. The closest Scots pine stands to the seed orchard are more than 500 m away.

### Material

Needles from multiple ramets of each of the 28 genotypes in the Västerhus orchard were collected to establish their genetic identity. Additional samples from 149 tree genotypes were collected in genetic archives and seed orchards across Sweden. These 177 trees are called plus trees because they are phenotypically selected from natural stands and make up a portion of the foundation of the Scots pine improvement program in Sweden (Stener et al. 2016). Seeds obtained from a bulk collection of cones produced by open pollination in 2014 in the Västerhus orchard, an additional orchard for diversity comparison and two unmanaged stands were germinated in a greenhouse at Skogforsk, Sävar, Sweden in the fall of 2016. The two unmanaged stands represent the approximate deployment area for the two sampled seed orchards, with Västerhus seedlings having the more southern deployment (Fig. 1). The plus trees and unmanaged stands together are expected to reflect the overall allele frequencies. Needles from 300 seedlings from each orchard and 50 from each unmanaged stand were harvested in early 2017. We also included haploid material from two Västerhus genotypes (eight megagametophytes each), representing selfed material and 29 samples from two congeneric species, *Pinus tabuliformis* and *Pinus yunnanensis*. In total, 922 samples were collected and genotyped.

### DNA isolation and GBS library prep

DNA was extracted from each seedling with an Omega Bio-tek E-Z plant kit (OMEGA Bio-Tek). GBS library preparation followed the procedure of Pan et al. (2015). Briefly, 200 ng DNA of each sample was digested with *Pst*I-HF^®^ (New England BioLabs^®^ inc.) and unique barcodes ligated with T4 DNA Ligase (New England BioLabs^®^ inc.) in a single reaction. The digested and ligated DNA of 300 individuals was pooled, purified, and PCR amplified. The purified PCR product was then separated on an E-gel^®^ Size-Select II™ pre-cast gel (ThermoFisher Scientific™), and fragments within the size range of 350–450 bp (including 125–132 bp adapters) were recovered. Paired-end sequencing (2 × 125 bp or 2 × 150 bp) was performed on an Illumina Hiseq2500 (SNP&SEQ Technology Platform, NGI Uppsala Genome Center) or Illumina HiSeqX (Novogene, Hong Kong). To test reproducibility within and between library preparations, 71 samples were replicated to a total of 160 replicates in the libraries to test reproducibility within and between library preparations.

### Genotype data filtering

Sequence reads were first separated by barcodes with Stacks: *process_radtags* (Catchen et al. 2013) into paired-end and single-end reads. The barcode sequences were removed from the sequences using Trimmomatic (Bolger et al. 2014). Sequences were then mapped to the *Pinus taeda* genome v1.01 (Zimin et al. 2014) with Burrows-Wheeler Aligner (BWA) (Li and Durbin 2009) and merged and sorted using SAMtools (Li et al. 2009) to produce a single BAM file for each sample. Several parameter combinations were examined to minimize error rates of replicates and maximize SNP overlap of samples to facilitate relationship estimation. Error rate was calculated as the proportion of allelic mismatches between replicates. The final filtering pipeline is as follows: samples are combined for variant calling using SAMtools—*mpileup*. Reads with minimum mapping quality of 10 (-*q* = 10) from the recalculated base alignment quality (-*E* option) are kept and then output as genotype probabilities (-*g* option). For SNP calling, we used SAMtools/BCFtools (Li et al. 2009) followed by VCFtools (Danecek et al. 2011) for filtering. Indels and SNPs within 5 bp of an indel were removed to avoid falsely called SNPs as result of indel misalignment. We excluded non-biallelic loci, genotypes, and bases with Quality <20 and genotype depth <8×. We also filtered for a minor allele frequency (MAF) threshold of 0.01 and scored SNPs if at least 60% of the individuals were represented (call rate 60%). As final filtering steps to diminish putative loci, which mapped to paralogous fragments, we removed loci with more than 70% heterozygous calls and loci that had both significant negative *F*_IT_-values (1-*H*_O_/*H*_E_) and were not in HWE. This step was examined using VCFtools with the—*hardy* option and filtered accordingly. We also examined the effect of filtering heterozygous calls when allele read depths deviated from the binomial.

We also performed a more rigorous filtering on the replicates by creating a reduced reference genome to map GBS-tags. The bam files were remapped to the reduced reference, and we used GATK indel realignment (GATK 3.8; Poplin et al. 2017) to produce more accurate alignments around indels. Hard filtering was first applied with GATK: QD < 2.0; FS > 60.0; MQ < 40.0; MQRankSum < −12.5; ReadPosRankSum < −8.0; SOR > 4.0, and then using VCFtools with the same parameters as above. Replicated samples were compared both for locus coverage and allele error rates for different parameter settings in SNP calling, and by the GATK and SAMtools filtering pipelines.

### Kinship analyses

We applied three methods to examine relatedness in the samples, namely genotype score correlation, the Ritland’s methods of moment (MOM) relatedness estimator (Ritland 1996) and Milligan’s maximum likelihood (ML) relatedness estimator (Milligan 2003). We expected to find four different kinship estimates: unrelated (relatedness coefficient = 0); half-sib, 0.25; full-sib and parent–offspring, 0.5; selfed and megagametophytes, 1.

Correlation between samples was calculated over the loci shared between two samples based on their genotype scores for those loci (0, 1, or 2). Ritland’s and Milligan’s relatedness estimator were calculated in the R-package “related” (Pew et al. 2015), which is an R implementation of the software COANCESTRY (Wang 2011a). Population allele frequencies were based on the set of 177 plus trees (28 Västerhus orchard parents and the 149 additional plus trees) and 100 natural stand samples in different combinations, as well as the congeneric species and all samples together to a total of eight reference sets (i.e., Västerhus parental genotypes, one or the other unmanaged stand, both unmanaged stands, all plus trees, all plus trees and unmanaged stands, congeneric species and finally the total dataset). The different combinations of samples were used as the allele frequency reference input for coancestry method in “related”. This was done to test the impact of the allele reference on relatedness estimates, because the variance of the relatedness estimator is expected to vary with the allele frequency reference according to Ritland (1996):

1 $$\mathrm{Var}\left( {r_{\mathrm{XYi}}} \right) = \frac{{s_i(1 - s_i)}}{{p_i^2q_i^2}},$$

where *s*_*i*_ is the expected similarity of two compared individuals based on the allele frequencies *p*_i_ and *q*_i_ of the *i*th locus. We compared different allele frequency references to establish an estimator with higher precision (more distinct relatedness status, i.e., lower variance). The allele frequency reference that gave results with the lowest variances was selected and used for further analysis. In this reference set, we removed all individuals showing elevated relatedness, thus assuming all remaining individuals are unrelated. If a large proportion of the individuals in the reference population are related, the relatedness estimates will be lower than expected (Wang 2014). All parent–offspring pairs were based on Ritland’s estimator and compared with the correlation and ML estimates. Only when all three estimates were in agreement (i.e., all three estimates gave the same top candidates) was the parent–offspring pair accepted. We used the established parent–offspring pairs to compare relatedness estimates of putative full-sibs and half-sibs which in turn was used to verify parental assignment of related seedlings.

To determine the optimum MAF threshold in relatedness estimations, we included samples representing different levels of kinship, e.g., unrelated trees, half-sibs and full-sibs, and estimated their correlation and Ritland’s relatedness estimator under MAF thresholds 0.01, 0.05, 0.1, 0.2, 0.3, and 0.4 with a bootstrapping-like scheme. We sampled a maximum of 1000 SNPs without replacement 1000 times for each comparison to build a 95% confidence interval. If there were <1000 loci to compare at a particular MAF, 75% of the available loci were used per sample run. This allowed us to estimate the MAF threshold that would give most power to distinguish between different levels of kinship in our dataset.

We also calculated the realized genetic relatedness matrix to compare established family structure with the estimated proportion of shared alleles (additive genetic relatedness) and genotypes (dominance genetic relatedness) between family members. The purpose was to evaluate whether GBS data would fit this method to quickly determine a relatedness matrix overall samples. The genomic relatedness matrix (G-matrix) was calculated according to VanRaden (2008) using the package “SNPready” (Granato and Fritsche-Neto 2018). The G-matrix calculations require a complete dataset, thus SNPs were filtered more rigorously by first removing samples that were missing more than 90% of the SNPs and then setting the call rate to 0.7 and MAF to 0.1. Missing values were imputed naïvely using population allelic frequencies and their probability of occurrence.

In addition to the above mentioned estimators used we explored the performance of six other estimators as well. Four (Li et al. 1993; Lynch and Ritland 1999; Queller and Goodnight 1989; Wang 2002) are included in the R-package “related” (Pew et al. 2015) and COANCESTRY (Wang 2011a). The last two we explored were v2 of NgsRelate, a maximum likelihood estimator of relatedness utilizing genotype probabilities rather than called genotypes (Hanghøj et al. 2019), and the KGD estimator which corrects for false homozygotes due to low sequencing depth (Dodds et al. 2015).

### Diversity measures

Genetic diversities in the form of average observed (*H*_O_), expected heterozygosity (*H*_E_), and MAF were calculated in R within each sample set. *H*_O_ was calculated by averaging the proportion of heterozygous individuals overall loci in the groups, *H*_E_ was calculated according to:

2 $$H_E = 1 - \frac{1}{m}\mathop {\sum}\limits_{l = 1}^m {\mathop {\sum}\limits_{i = 1}^k {p_{li}^2} }$$

where *p*_li_ is the allele frequency for the *i*th allele of *k* possible alleles (we only used biallelic loci) at the *l*th of *m* loci

For the Västerhus orchard seedlings, we estimated the effective number of parents (*N*_ep_) from the reproductive success among the parents based on the function of effective number of types (Simpson 1949) using the sampling-bias corrected estimate (Nielsen et al. 2003).

3 $$N_{\mathrm{ep}} = \frac{{\left( {n - 1} \right)^2}}{{\mathop {\sum }\limits_{i = 1}^{\mathrm{Np}} p_i^2\left( {n + 1} \right)\left( {n - 2} \right) + 3 - n}}$$

where *n* is the number of seedlings sampled, Np is the number of parents, and *p*_i_ is the relative reproductive success of the *i*th parent. We calculated the estimate with and without external pollen sources. We estimated the frequencies of each external pollen source through the relatedness of seedlings with at least one unassigned parent.

## Results

### GBS error rate and reproducibility

Examination of the genotypes after filtering with a median depth of 41 for a supported call (mean 67.3) of replicated samples showed that the error rate (average percent of alleles that were not consistent over replicates) was around 5% (Fig. S1). This error rate was used in the calculation of the likelihood relatedness estimator. The difference in error rates between the two data processing pipelines was small, on average 3.7% lower for the GATK pipeline (GATK_e_ = SAM_e_ 0.963, *R*^2^ = 0.9) giving an error rate of around 4.8%. The final SNP calling on the total dataset was produced with the SAMtools pipeline as the more memory and time demanding GATK pipeline offered only slight improvements. The proportion of shared loci between replicates varied substantially, from 0.55 to 0.99, but the overall median was high, 0.89 (Fig. S2). The median proportion of shared loci for replicates within libraries was 0.90, and 0.87 between libraries.

### Kinship reconstruction

We choose to use the combination of the 177 plus trees and the two unmanaged natural stands as allele frequency reference for the relatedness estimator because we observed lower variance in the estimate (maximum peak height in the 3D-density plot, Figs. S3 and S4) with these samples as reference. For the final allele frequency reference set, we removed seven genotypes from the natural stands due to pairwise elevated relatedness and one sample due to low coverage. We also removed six plus trees due to elevated relatedness (these trees come from controlled crosses of other plus trees) and six trees with lower coverage (<80%). We observe a unimodal distribution of the relatedness estimate of non-related individuals suggesting a uniform allele frequency of the seedlings and parental trees (Fig. S4). We examined the population structure of the selected reference set and found that only a small fraction of the genetic variation (≈0.22%) can be attributed to genetic structure and/or library effects (Fig. S5).

**Fig. 2:**
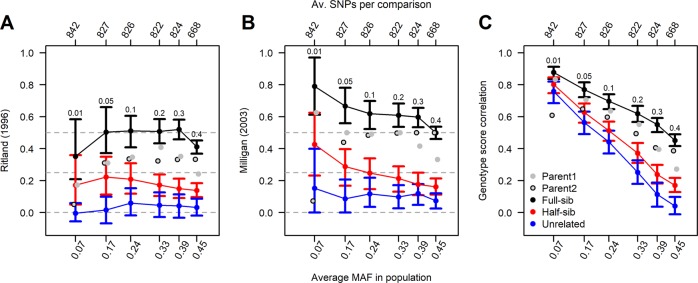
Pairwise comparison of three seedlings with assumed relationship to seedling 8P16-14-2 for Ritland’s, Milligan’s ML estimator and genotype score correlation for MAF 0.01, 0.05, 0.1, 0.2, 0.3, and 0.4. The *x*-axis shows the average minor allele frequency in the population after filtering for the various thresholds. Numbers above the second *x*-axis is the average number of SNPs in the population representing the subsampled loci. We sampled 1000 SNPs for 1000 times per MAF cut off except for MAF 0.4 to get an estimated 95% confidence interval. Numbers above the top C.I. bars are the MAF cut off.

Allele frequency distribution overall Scots pine samples showed that rare-allele SNPs were the most common and around half of all the SNPs had a 0.01 ≤ MAF ≤ 0.05 (Fig. S6a). The minor allele frequencies are relevant for the power of separating different relatedness. Higher MAF SNPs have the most power but are uncommon and the opposite is true for MAF at the low range. This is visible in our relatedness estimates (Fig. 2). With low MAFs (e.g., 0.01) the relatedness estimates show low precision and the genotype score correlation is large (Fig. 2). This is the result of the properties of the allelic frequency spectrum where the majority of the genotypes are the same (homozygous for the common allele, Fig. S6a). The power of separating different relatedness increases with increasing MAF, but the number of available SNPs for comparison decreases rapidly (Fig. S6b). With lower numbers of SNPs, the discrimination power between relatedness decreases (Fig. 2). We thus set the MAF threshold to 0.1 to retain as many SNPs (7387, see Fig. S6b) as possible for inferring relatedness.

**Fig. 3:**
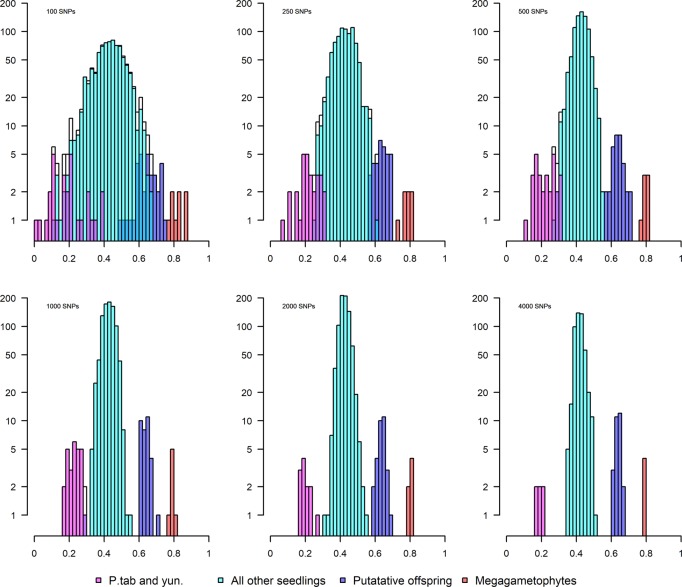
Recovery of different levels of kinship under different no. of SNPs. *Y*-axis is the log no. of individuals and *X-*axis is pairwise genotype score correlation between samples. The results show pairwise comparison between Västerhus parent AC3065 and 4 categories of samples: megagametophytes (haploid) from AC3065; putative offspring of AC3065; all other samples of Scots pine including natural stand seedlings, additional orchard crop and all other plus trees; and samples of two congeneric species *P. tabuliformis* and *P. yunnanensis*. MAF = 0.1 was applied to all comparisons.

With the MAF threshold set to 0.1, we examined the discrimination power by varying number of SNPs from 100 to 4000. In the example shown in Fig. 3, one parent is compared pairwise to all other samples included in the study to estimate the putative relatedness. We found that different levels of relatedness were easily separated above 1000 SNPs (Fig. 3), and thus based our assessment of relatedness on comparisons, which included at least 1000 SNPs. We also found that the pairwise comparisons among *P. sylvestris* samples had a unimodal distribution indicating an absence of genetic structure and a uniform allele frequency spectrum across the range, at least at MAF 0.1 (Fig. S5).

**Fig. 4:**
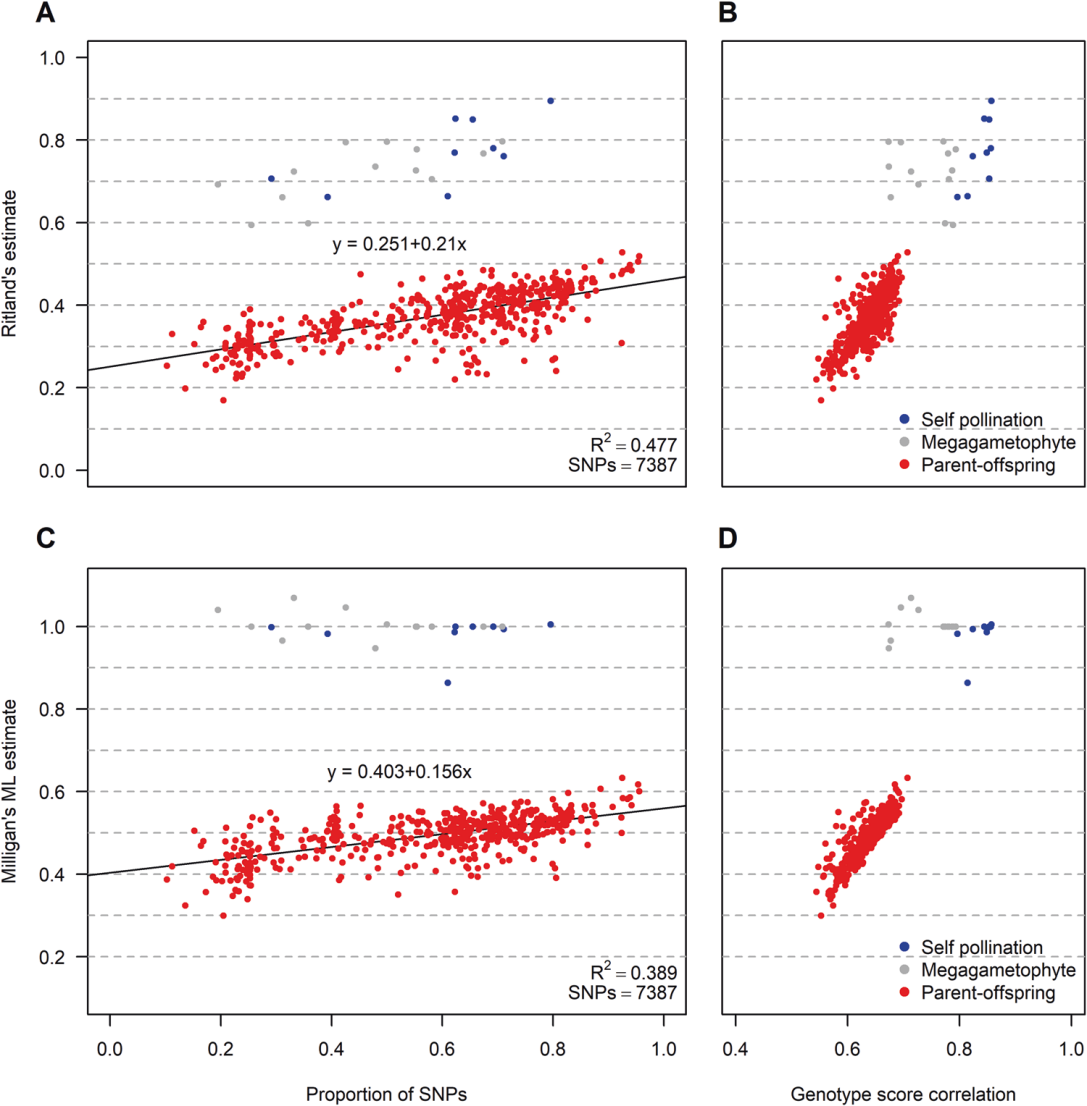
Four scatter plots of pairwise relationship estimates of Västerhus seedlings and the genotypes assumed to be their parents. Blue filled circles are detected self-pollination, gray are haploid megagametophytes, and red are those deemed to be parent–offspring. **a** Ritland’s estimate as a function of the shared proportion of SNPs that could be used for the comparison, total number of SNPs after filtering (0.1 MAF, allowed for 40% missing, and loci with 70% heterozygosity or less, loci with significant negative *F*_IT_-values were removed) was 7387. **b** Ritland’s estimate as a function of the correlation in genotype scores. **c** and **d** similar to **a** and **b**, respectively, but with Milligan’s ML estimation of relatedness.

In addition, we examined how the differences in heterozygosity filtering and the proportion of SNPs available for comparison affected the relatedness estimate. More rigorous heterozygosity filtering (the maximum percentage of heterozygous genotypes allowed for a locus to be kept) improved the relatedness estimate slightly, although filtering for negative *F*_IT_-values appeared to be more effective, especially for Milligan’s ML estimation method in conjunction with correcting for the error rate (Fig. S7). The proportion of SNPs available for a comparison had, however, the largest effect on the tested methods. The parent–offspring relatedness estimate using Ritland’s method went from 0.28 when 20% of the total number of SNPs were available for the comparison to 0.43 when 80% of the SNPs were shared among the two compared samples. These estimates came closer to the expected 0.5 with the ML-method, with an estimate of 0.43 at 20% shared SNPs up to 0.53 at 80% (Figs. 4. and S8).

Among the 300 seedlings from the Västerhus orchard crop, two samples had very low coverage and were discarded from analyses. The pairwise comparisons between the 298 offspring and 28 parents had an average of 4326 shared SNPs (average of around 82% loci overlap between parent and offspring, Table 1) and a standard deviation of 1447 SNPs at MAF 0.1 and 40% missing. All pairwise relatedness established by the three estimators agreed well, Furthermore, the estimator of Milligan (2003) was less sensitive to low proportions of shared SNPs between sample pairs, indicated by the flatter slope (Figs. 4 and S9). The Milligan’s estimate also showed clear distinction between parent–offspring pairs and self-pollination events and megagametophytes. Filtering away erroneous heterozygous calls effectively increases the relatedness estimators and decreases the dependency on shared SNPs between samples. We see almost no effect of removing heterozygous genotype calls due to deviation from the binomial (data not shown). Removing loci with more than 70% heterozygotes had some effect while removing loci with negative *F*_IT_-values was most effective together with higher MAF (Figs. 2, S8, and S9). The tested KGD estimator compensates for varying sequencing depths of genotype calls (Dodds et al. 2015). Some of the observed dependency between the proportion of shared SNPs and relatedness estimates lie in the varying depths of these samples, although other unknown factors may also distort the results (Fig. S9). The parent–offspring pair assignment by the MOM and ML estimators also had the greatest genotype score correlations relative to other comparisons (see Figs. S4 and 4 for comparison between correlation and Ritland’s estimate). Parental assignment was successful for 295 of 298 seedlings, and the remaining three could not be assigned to any of the parental genotypes in the orchard.

**Table 1:** Summary of Scots pine samples (*N*) included in the study, their sequencing depth, no. of SNPs and standard deviation (SD), and genetic diversity in the form of observed (*H*_O_), expected heterozygosity (*H*_E_) and average minor allele frequency (MAF).

Samples	N	Median depth	Average no. SNPs	SD	*H*_*O*_	*H*_*E*_	MAF
Parents	28	14	5256	1248	0.357	0.338	0.365
Offspring	298	44	6375	1069	0.338	0.342	0.367
Nat. stands	99	44	6762	606	0.337	0.34	0.366
Add. orchard	300	16	5161	1299	0.312	0.332	0.359
Plus trees	149	19	5090	1517	0.225	0.328	0.345

Västerhus orchard parents, offspring, natural stands, a crop from an additional seed orchard, and a group of Scots pine plus trees selected across Sweden were included in this analysis.

### Diversity and mating structure

Based on the parent–offspring assignments, selfing rate was low in the Västerhus crop; only 9 seedlings were identified as being the result of self-fertilization or crosses between clone mates (3%, see Fig. 4), and 39 crosses were replicated into full-sib families with 2–6 offspring each. A total of 98 seedlings could be assigned to only one parent from the orchard and 196 (including selfing) had both parents from the orchard. This resulted in an estimated 33.6% pollen contamination.

Reproductive success varied among the parents in the orchard. Based on their ramet abundance, most parents produced fewer offspring than expected, while a few were highly overrepresented (Table 2), which is likely to be correlated to the genotypes’ pollen production (Torimaru et al. 2012). The number of internally and externally pollinated seedlings varied little among parents. Parents that produced a large proportion of internally pollinated seedlings also produced a large proportion of the externally pollinated seedlings (adjusted *R*^2^: 0.84, *P* value ≪ 0.01). Parent Z4019 produced five externally pollinated seedlings of a total of 16, which is a larger proportion than other parents produced (Table 2).

**Table 2:** The number of seeds produced by internal (IP) and external pollination (EP) by each Västerhus orchard parent and the expected number (E(IP), E(EP)) based on their relative abundance (ramet number) in the orchard.

	Parent ID	No. rametes	IP	EP	E(IP)	E(EP)
	AC3056	332	30	4	34.4	8.6
	Y2005	309	28	7	32	8
	Y4016	276	24	8	28.6	7.2
	AC1006	239	9	2	24.8	6.2
	Y3012	229	11	2	23.7	5.9
	Z3007	221	27	5	22.9	5.7
	Y4507	214	48	15	22.2	5.5
	Z2081	206	39	9	21.4	5.3
	Y3014	192	36	14	19.9	5
	AC2064	167	15	3	17.3	4.3
	Y3001	166	14	5	17.2	4.3
	AC3040	127	18	2	13.2	3.3
	Z4022	122	7	1	12.6	3.2
	Z4003	108	7	1	11.2	2.8
	Y4508	107	15	5	11.1	2.8
	Z3029	93	7	0	9.6	2.4
	Z3009	87	4	0	9	2.3
	X4203	67	1	0	6.9	1.7
	AC4221	65	9	3	6.7	1.7
	Y2004	64	3	1	6.6	1.7
	Z4019	60	11	5	6.2	1.6
	AC1075	58	2	0	6	1.5
	AC2047	55	3	0	5.7	1.4
	AC3033	49	2	0	5.1	1.3
	AC3015	47	3	1	4.9	1.2
	AC3065	46	3	0	4.8	1.2
	Y4103	41	3	1	4.2	1.1
	Z4032	35	13	4	3.6	0.9
Total	28	3782	392	98		

**Fig. 5:**
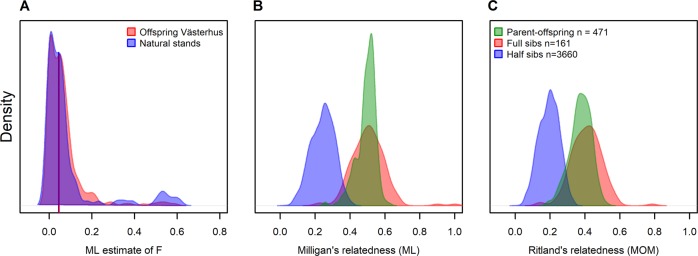
Density plots of individual inbreeding coefficient and relatedness estimates. **a** Milligan’s ML of individual inbreeding coefficient of the Västerhus crop (median = 0.047) and the two sampled natural stands (median = 0.045). **b** Distribution Milligan’s pairwise relationship estimates of parent–offspring (green, point estimate = 0.52), full-sib (red, 0.51) and half-sib pairs (blue, 0.26) as well as the distribution of (**c**) the Ritland’s estimates (0.37, 0.42, and 0.20, respectively). The numbers in the legend of **c** represent the number of pairwise comparisons made in each category.

Including the background pollen contamination as a source of unique pollen donors, the estimated effective number of parents *N*_ep_ was 23.2, and excluding externally pollinated seedling in the calculation resulted in an estimate of 16.2. The observed average heterozygosity (*H*_O_) was higher than the expected (*H*_E_) for parental trees, similar to that observed in natural stands and the other orchard crop, and slightly lower than expected for the additional plus trees (Table 1). However, the individual inbreeding coefficient (*F*) of the Västerhus orchard crop and the natural stands showed very minor differences. The Västerhus orchard crop had a slight shift toward greater individual inbreeding coefficients compared with seedlings from natural stands (Fig. 5a), with a median of 0.047 and 0.045 for Västerhus crop and natural stands, respectively. Estimates of relatedness between seedlings produced by self-pollination and their parents compared with megagagametophytes and their parents were similar although the genotype score correlations between the megagametophytes and parent appear to be lower (Fig. 4a–d). The likelihood point estimates of relatedness for parent–offspring pairs (0.52) and full-sibs (0.51), were close to the expected 0.5. The half-sib pairwise comparison was also close (0.26) to the expected value (0.25, see Fig. 5b). The Ritland estimates, on the other hand, had lower than expected values, especially for parent–offspring (0.37) but also for full-sib (0.42) and half-sib (0.20, see Fig. 5c) pairs.

A G-matrix provides an overview of the proportion of shared alleles among the samples, and is expected to follow estimates based on pedigrees. The G-matrix calculations require a full set of all samples comparisons to determine the proportion of shared alleles between samples. The analysis was thus based on more rigorously filtered data; 862,176 data points were inputed (17.53%) for a final number of 5028 SNPs for the analysis. The G-matrix relationship estimate is based on alleles shared among samples and reflects the proportion of additive genetic covariance. The dominance G-matrix reflects the shared genotypes and is proportional to the shared dominance genetic covariance among samples. The G-matrices estimated from our samples reflected kinship status, although the variation in estimation from shared alleles (relatedness estimates, proportional to the shared additive genetic covariance, *A*_R_ ∝ $\sigma _A^2$) and shared genotypes (proportional to the shared dominant genetic covariance *D*_R_ ∝ $\sigma _D^2$) was large within the different relatedness classes and with a lower average than expected. For parent–offspring, the median *A*_R_ was 0.22 with a standard deviation of 0.091 (*D*_R_ = 0.027 ± 0.047), slightly larger for full-sibs, 0.29 ± 0.086 (*D*_R_ = 0.15 ± 0.068), and 0.14 ± 0.049 for half-sibs (*D*_R_ = 0.025 ± 0.00.026).

To visualize the differences in relatedness estimations, we reconstructed the extended family structure for all the offspring from one parent (Y2005) and its mates using Ritland’s estimator (Fig. S10a). The pairwise relatedness among the individuals was also presented as a tree (Fig. S10b) where branch length reflects genetic distance, mirroring the relatedness between different sub-families. The G-matrix for these samples is pictured as a heatmap of the shared additive and dominant genetic variation in Fig. S10c, Supporting information. The clear distinction between parent–offspring observable in the Ritland’s estimator is not discernable in the G-matrix, but the overall pattern is reflected.

## Discussion

### GBS filtering

GBS is a cost-effective method to assess genetic variation of populations. However, due to the random distribution of coverage across samples and GBS libraries, missing data creates problems. In this study, orchard parents and offspring were analyzed from different GBS library preps and different sequencing systems (Illumina HiSeq 2500 and HiSeq X), which introduced some reduction in overlapping fragments between runs. However, a larger source of noise probably arises from the difficulty of manually sampling the same fragment length distribution accurately among libraries during the GBS library preparations. Along with the inherent errors from the GBS method, e.g., genotyping error and allelic dropout, this requires a filtering and relatedness estimation strategy that handles these sources of noise adequately (Attard et al. 2018; Wang 2017). The reference guided mapping procedure applied to our data along with our final filtering procedure reduced the allelic dropout among replicates from 1.2 to 0.1% (different homozygote alleles between replicates, data not shown) but the error rate remained around 5%.

This error rate (5%) with a minimum coverage threshold of 8× is expected from the GBS method when using reference alignment (Fountain et al. 2016; Lou et al. 2013). Errors may be derived from null alleles, where a mutation removes the digestion site for one or both strands in an individual, or allelic dropout from PCR reactions. However, the effect of null alleles for parental assignment when implementing the Ritland (1996) estimator is small with a large number of markers (Wang 2007). Some relatedness estimators are influenced by rare alleles (Wang 2011b), which in turn can be a consequence of allelic dropout, but setting the MAF threshold at 0.1 is expected to eliminate most of those loci and we observe a stabilization of our estimates at that threshold (Fig. 3).

In many instances the computational resources can be a limiting and expensive factor for bioinformatic pipelines especially for organisms, such as conifers, that have large genomes. We could effectively assign parent–offspring pairs using a less resource-demanding pipeline employing BWA for mapping, and filtering with SAMtools and VCFtools. The latter tools handle memory assignment better when dealing with large reference genomes compared with JAVA-based tools like GATK. Low depth sequencing of a large number of individuals allows confident parental assignment when incorporating the established error rate and basic filtering, at least in Scots pine.

### Estimation of kinship

For pairwise relatedness estimates, a reliable allele frequency reference and good coverage of the samples are needed. Loci with higher MAF are more informative but are rare, and a MAF of 0.1 retained enough SNPs to allow for a low call rate and still establish reliable allele frequencies for use in pairwise comparisons (Fig. 2).

All Västerhus orchard genotypes in this study are well tested plus trees, selected for their phenotype in natural stands, and are unrelated. With no external pollen contribution, the Västerhus orchard crop should only have four classes (five including unrelated) of relatedness: parent–offspring, full-sib (first degree relatedness), half-sib (second degree relatedness), and a few seedlings produced by self-pollination. The three methods of relatedness estimation agreed well in all pairwise assignments, although the estimated degree of relatedness varied substantially within each class of relatedness (Fig. 5b, c). This variation is expected for full- and half-sibs and arises from the stochastic processes of recombination (Attard et al. 2018; Powell et al. 2010). Although parent–offspring have lower variance, the expected variance is zero because offspring should share exactly one allele at each locus from each of its parents. Distant relatedness between parents could increase relatedness estimates of parent–offspring pairs and in turn the variance around the estimate, but not to the extent observed. We therefore conclude that the variance observed mainly originates from an unknown source, possible erroneous calls. The variance around relatedness estimates is inversely proportional to the number of markers used (Yang et al. 2010). This study utilizes comparatively few SNPs and thus precision of relatedness estimates are likely to be low relative to whole-genome sequence data. Further, when coverage and depth are comparatively low for any one sample of GBS data in the pairwise comparisons, the relatedness estimate is lower than expected (Fig. 4; Dodds et al. 2015), while estimates from SNP arrays with similar SNP numbers are closer to expected values (Munoz et al. 2014). This suggests that samples with less coverage have a lower proportion of quality SNPs (Fig. S5), which in turn leads to an underestimate of the relatedness. The main source of these low quality SNPs is often improperly called heterozygous SNPs (Patel et al. 2014). In line with this, erroneous heterozygous calls observed in our haploid megagametophytes were scored with high genotype quality by the caller algorithm, and these heterozygous sites differed among the haploid samples (data not shown). That is, although the mapping software calls a heterozygous SNP with high probability, it may be a false positive. Stricter filtering of heterozygosity rates led to slightly increased relatedness estimates although the effect was much lower than for coverage (Fig. S5). One reason for this could be strand bias, which results in differently called SNPs from a sample depending on which strand was sequenced. This phenomenon is difficult to filter because there is no consistency in which loci are affected and it is not limited to any one mapping method (Guo et al. 2012). On the other hand, trying to correct for paralogous calls and account for genotyping errors improved the estimates substantially. One possible reason for this is because the genotyping of the parental trees appeared to suffer more from paralogous calls than other sample categories, as suggested by the elevated *H*_O_ (Table 1). This, in turn, could also indicate the presence of contamination and/or degradation of DNA in these samples (Graham et al. 2015).

In Scots pine, pairwise genotype correlation performed well in distinguishing simple relatedness (parent–offspring and full-sib), and was strongly correlated with Ritland’s estimate (Fig. 3), although fine adjustment of thresholds was necessary for samples with lower coverage (Figs. 4 and S5). It will be more difficult to infer relatedness in an orchard crop where varying degrees of relatedness are already present among the orchard parents’ genotypes, especially with GBS data. For such instances, greater coverage or higher sequencing depth is necessary, and the length of IBD blocks used to estimate the number of crossover events between individuals must be established to better infer pedigree and relatedness status in a crop (Powell et al. 2010; Speed and Balding 2015).

The coefficients of relatedness estimated from the method of moments in this study are based on the assumption that the scored genetic variants of the samples are correct and that any genotyping errors will reduce the relatedness estimates. One way to mitigate this reduction is by using a maximum likelihood method that can correct for genotyping and/or genotype call error rates and constrain the estimates to biologically meaningful values (see Figs. S7 and S9; Milligan 2003). This would not necessarily make pedigree reconstruction easier but relatedness estimates would be more accurate. The proportion of shared genetic information between individuals is most often visualized in a G-matrix. Building a G-matrix and assigning the proportion of shared additive and dominance genetic covariance among individuals requires that all SNPs are scored and that missing data points are imputed for estimation purposes. Low-coverage GBS data are not suitable for this type of analysis if a large proportion of data points need to be imputed, especially in a species where genetic population structure and linkage disequilibria are insignificant, providing little guidance to the imputation step. The example presented in Fig. S10C shows some discernable pattern of increased proportion of shared alleles within families although not to the extent of what was expected, e.g., the two seedlings that appear to be a product of selfing shared more alleles (0.315 and 0.318, respectively) with all other offspring than with their parent (Y2005, 0.213). However, the dominant genetic relatedness shared between seedlings from self-pollination and parent was high, as expected. When compared with the average Ritland estimate of 0.42 for full-sibs, which was slightly lower than expected (Fig. 5c), the proportion of shared alleles (*A*_R_) among full-sibs was 0.29 while only 0.21 for parent–offspring, well below the expected 0.5. This could be due to the lower coverage of the parental samples (Table 1), and thus more imputations for those samples. Imputation based on population allelic reference (i.e., naïve imputation) will diffuse the signal of pairwise relatedness because individual differences are important in such analyses. We assume that the naïve imputation can enhance the effect of erroneous heterozygous calls (especially if these are in the allele reference population) on the underestimation of relatedness estimation. One way around this would be to first establish the pedigree and then to use that as a guide to the imputation step similar to that used in nearest neighbor imputation methods (Wang et al. 2012). However, the expected outcome would still be values lower than expected for the same reasons that most relatedness estimators are lower than expected.

### Diversity

The genetic diversity of our sampled individuals is high and levels of genetic structure are low. The distribution of minor allele frequencies (Fig. S3) show “L”-shaped patterns similar to other conifers with wide distribution ranges (Chen et al. 2016; Conte et al. 2017). Both the MAF distribution and that very low genetic differentiation is found in the PCA of the allele frequency reference population (Fig. S5) indicate a large population with near random mating structure maintaining high genetic diversity.

Genetic diversity in the Västerhus orchard crop where all parents are unrelated remains high compared with natural stands (Table 1), although the average individual inbreeding coefficient is slightly higher (Fig. 5a). The linear deployment strategy increases the genetic contribution from the most common parent genotypes to the crop even though flowering capacity and phenology differences among parents can modify the expected genetic makeup of the crop. Previous studies in this orchard have estimated the effective number of fathers in seeds collected from known mother trees, with an average of 13.27 ranging from 9.31 to 15.92 (Funda et al. 2016; Torimaru et al. 2012, 2013). In this study we made a comparative estimate, but because we cannot identify the pollen donor we estimated the effective number of parents instead. This estimate was 23.2 when external pollination events were included, and 16.2 excluding external pollen contamination, which is close to the largest previous estimate excluding pollen contamination (Funda et al. 2016). Even when pollen contamination was included, the effective number of parents was still well below the number of actual parents (28) represented in the orchard, suggestive of a departure from panmixia. Previous investigations in the orchard have shown large differences between parents in flower production, and the parents that produced the most seedlings in this study have been shown to have a larger pollen production (Funda et al. 2016; Torimaru et al. 2012).

Some of the seedlings classified as sired by external pollen could have been produced by rootstock within the orchard. There were 67 overgrown rootstocks in the orchard, which make up 1.8% of the ramets in the orchard. Bulk collected seeds in the orchard likely carry genetic representation of the rootstocks.

The inbreeding coefficients in the Västerhus seedlings were slightly higher than those observed in the natural stands (Fig. 5a). The increased number of individuals with an inbreeding coefficient above 0.2 (right hand tail of Fig. 5a) implies that some seedlings, 8.1% from natural stands and 3% from Västerhus orchard parents, are the products either of selfing or of mating between related individuals. The proportion of self-pollination found in Västerhus is close to the expected value of 3.3%, given genotype frequencies and the level of external pollen contribution to the crop

4 $$E(f_s) = \left( {1 - \mathrm{PC}} \right){\sum} {P_i^2},$$

where *E*(*f*_*s*_) is the expected proportion of seedlings from self-pollination, PC the proportion of pollen contamination, and *P*_i_ is the frequency of genotype *i* in the orchard (Table 2).

The seedlings with elevated inbreeding coefficients are also identified as a product of selfing in the relatedness estimation (Fig. 4). The seeds produced in natural stands are not expected to travel long distances and relatedness among adjacent trees is expected to be elevated creating family structure (García-Gil et al. 2015). Family structure is not expected in seed orchards where parents are unrelated, planted randomly, and new seedlings are not allowed to establish. However, ramets die and family structure might arise in the orchard if seeds chance to germinate and establish near the dead ramet. In the Västerhus seed orchard, some ramets categorized as overgrown rootstock may actually be seedlings produced from within the orchard which in turn could produce seedlings with higher inbreeding coefficients.

## Conclusion

This study illustrates that the GBS method can be effectively applied in kinship estimations in Scots pine. Under our optimized data processing procedure, relatedness, and genetic composition, including level of pollen contamination within a seed orchard crop, can be established consistently by different estimators. Correcting for putative paralogous mapping by excluding excessive heterozygous loci and accounting for genotyping error rates substantially improved the reconstruction of relatedness, particularly for samples with lower coverage. However, the results show that more must be done to optimize filtering strategies and to increase the understanding of how GBS data behaves in different applications. In orchard crops there are only three classes of relatedness (disregarding self-pollination events and unrelated samples) to establish, and for these estimates no major difficulties were encountered even though manual corrections of thresholds were needed, especially in cases with low overlap of SNPs between samples. However, the situation may become more challenging in natural populations with continuous and more complex pedigree structures.

Determining the genetic composition and diversity of breeding, and natural populations and reforestation materials are invaluable for keystone species in boreal forest ecosystems. This is also of economic relevance for forest owners to forecast the breeding gain of their forests. Estimating relatedness from genomic data also has important evolutionary applications, e.g., in conservation programs, and for improving the accuracy of heritability estimates of target traits. Furthermore, studies of orchard trees can aid estimation of effective gene flow for particular forest tree species. The level of external pollination should be a good indicator of long distance pollen dispersal in a species, an estimate that has been difficult to obtain in natural populations (Robledo-Arnuncio 2011). In light of the low genetic differentiation in *P. sylvestris* across the entire sampled range, we expect pollen flow to be extensive and thus, all else being equal, pollen contamination rates will be higher in comparison to seed orchards of other conifer species.

## Supplementary information

Supplemental Material

## Data Availability

The data and scripts underlying the manuscript have been deposited with the Dryad repository. The VCF genotype file, vcf filtering procedure, RData and R scripts, and information on how to reproduce the results has been deposited at Dryad: 10.5061/dryad.h44j0zpg5.
