# Supplementary material for: Parentage and relatedness reconstruction in Pinus sylvestris using genotyping-by-sequencing
Source: Heredity (Edinb). 2020 Mar 2;124(5):633–46. doi: 10.1038/s41437-020-0302-3 (PMC7171117; doi:10.1038/s41437-020-0302-3)
Supplement: Supplementary file 1 — Supplemental Material [file 41437_2020_302_MOESM1_ESM.pdf]

Supporting information for Hall D, Zhao W, Wennström U, Andersson Gull B and Wang XR

Parentage and relatedness reconstruction in *Pinus sylvestris* using genotyping by sequencing.

Supporting information

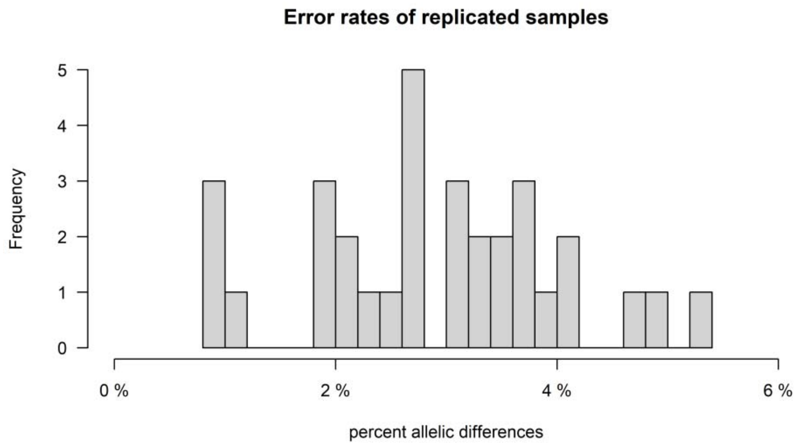

Figure S1. Allelic differences between replicates.

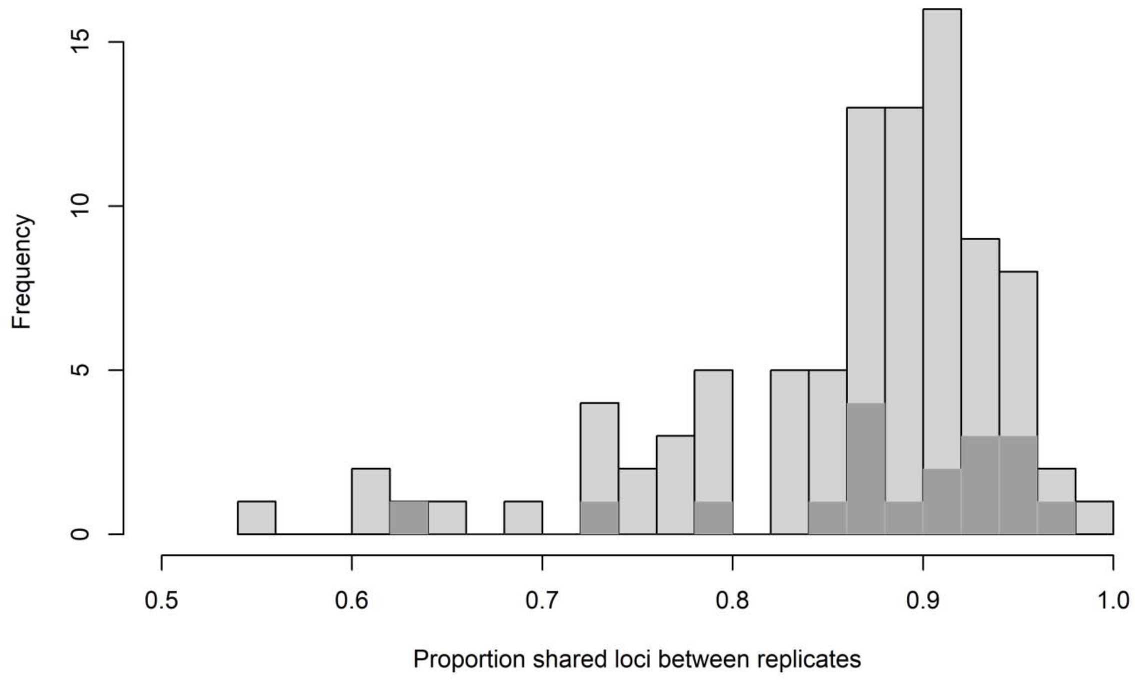

Figure S2. Histogram showing the proportion of shared loci between replicates based on the sample with the lowest coverage, between and within libraries, median = 0.89. Darker portion of bars represents replicates within libraries, median = 0.90.

Supporting information for Hall D, Zhao W, Wennström U, Andersson Gull B and Wang XR

Parentage and relatedness reconstruction in *Pinus sylvestris* using genotyping by sequencing.

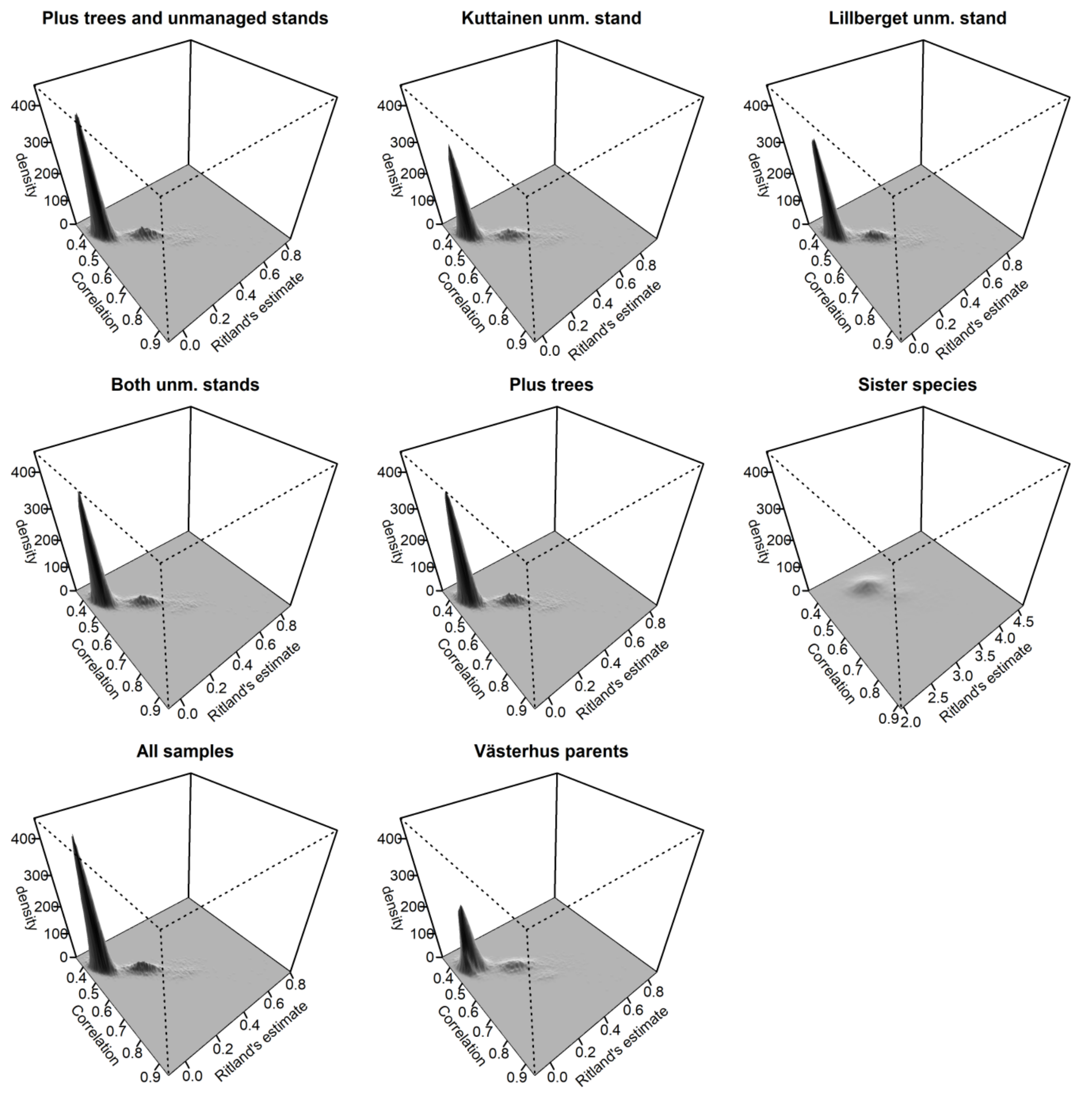

Fig. S3. Three dimensional density plots of all Västerhus parents and offspring pairwise comparisons (53 628) for genotype correlation and relationship Ritland (1996) based on different reference population allele frequencies (see plot titles). These were made in order to test the impact of different reference sets on the variance of relatedness estimates. The top left reference population (N=257) uses only unrelated trees sampled across Sweden, the biologically reasonable population reference to catch population allele frequencies. Unmanaged stands as reference: Kuttainen N=44; Lillberget, N=48 and both N=92. Plus trees, N=164; Congeneric species, N=29; All samples (excluding congeneric species), N=893; and finally the Västehus parental plus trees, N=28. There appears to be a larger variance of estimates of unrelated pairs with decreasing numbers of individuals in the reference set although the accuracy do not change, except when allele frequencies are very different such as using congeneric species as a reference set.

Supporting information for Hall D, Zhao W, Wennström U, Andersson Gull B and Wang XR

Parentage and relatedness reconstruction in *Pinus sylvestris* using genotyping by sequencing.

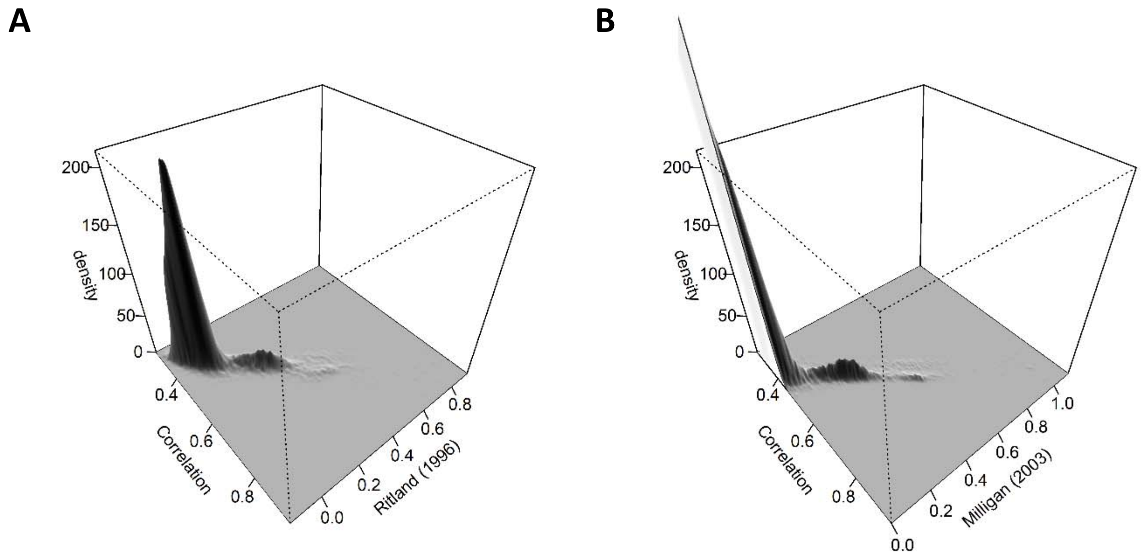

Figure S4. 3D-density plot that shows the correlation between the Ritland (1996) estimator and the pairwise genotype correlations of all Västerhus seedlings and parents. **B)** Similar to **A** but with Milligan's ML estimation of relatedness instead.

Supporting information for Hall D, Zhao W, Wennström U, Andersson Gull B and Wang XR

Parentage and relatedness reconstruction in *Pinus sylvestris* using genotyping by sequencing.

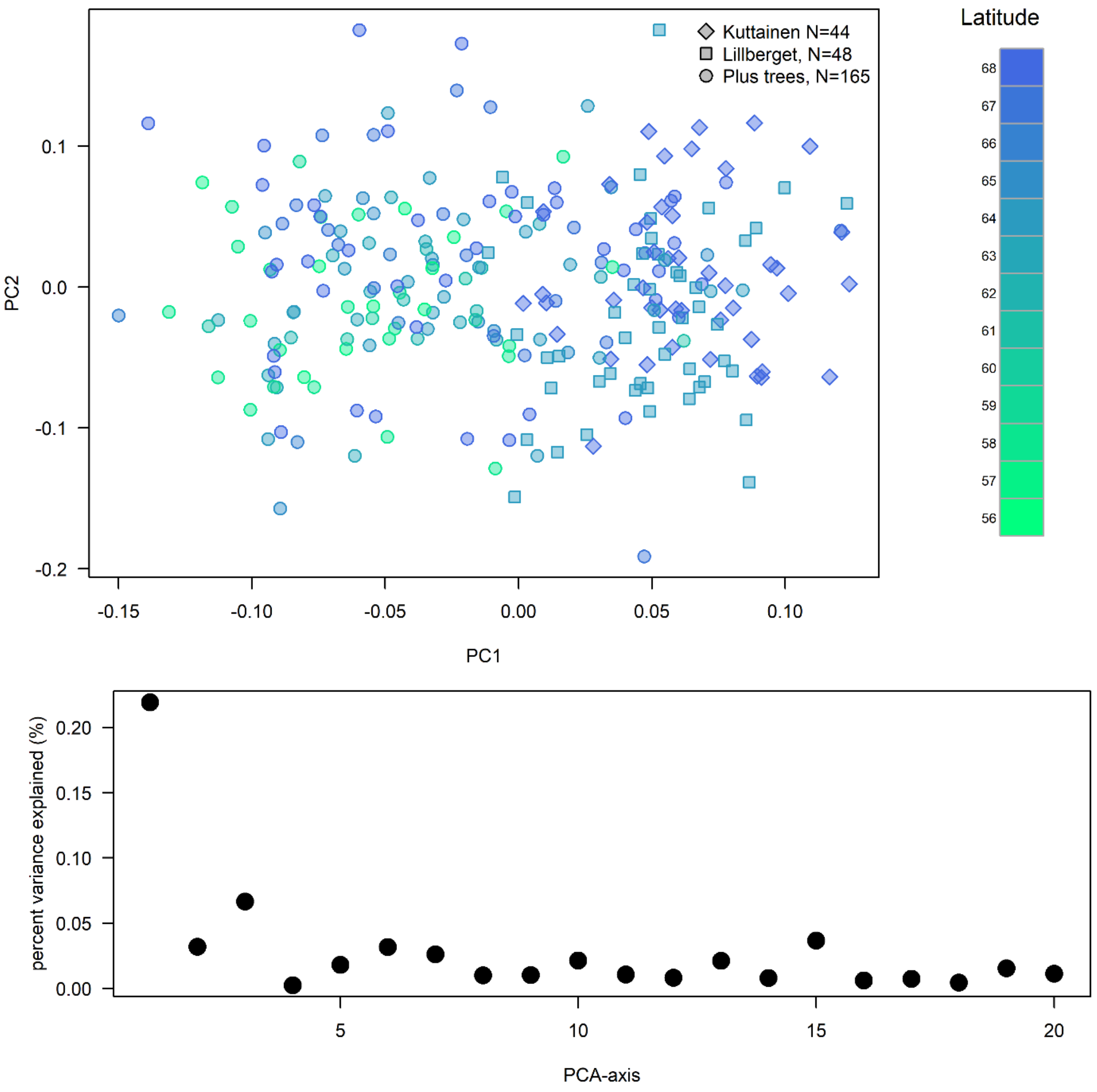

Fig. S5. Top plot is a PCA of the plus-trees and natural stand samples making up the allele frequency reference colored according to their origin. Bottom plot shows the genetic variance explained by each of the 20 first PCA-axes, where the first axis explain about 0.22% and the remaining ≈0.05% each. PCA was calculated using the pcadapt package in R (Luu *et al*, 2017) allowing for only 10% missing. There is no imputation step, but to account for missing data, the correlation matrix between individuals is computed using only the markers available for each pair of individuals and then the Mahalanobis distance between samples is calculated.

Supporting information for Hall D, Zhao W, Wennström U, Andersson Gull B and Wang XR

Parentage and relatedness reconstruction in *Pinus sylvestris* using genotyping by sequencing.

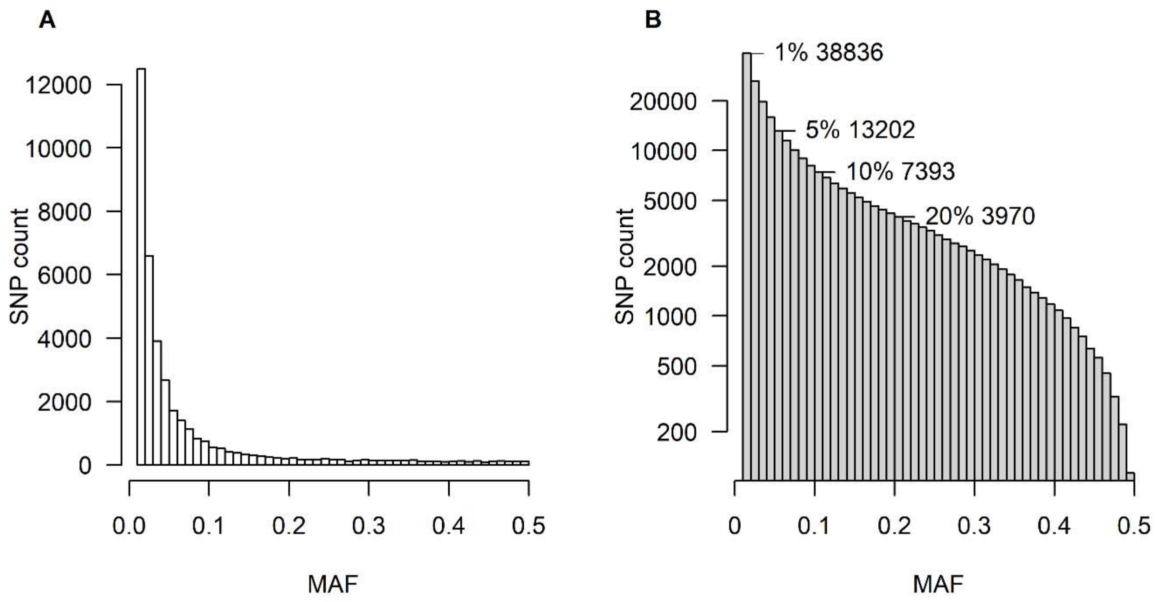

Figure S6. The distribution of SNPs according to their MAF (Minor allele frequency, **A**), and the total no. of SNP remaining at different MAF threshold (**B**). Note: 7393 SNPs at MAF 0.1 with congeneric species included and 7387 when excluded (i.e. Fig. 4 in the manuscript).

Supporting information for Hall D, Zhao W, Wennström U, Andersson Gull B and Wang XR

Parentage and relatedness reconstruction in *Pinus sylvestris* using genotyping by sequencing.

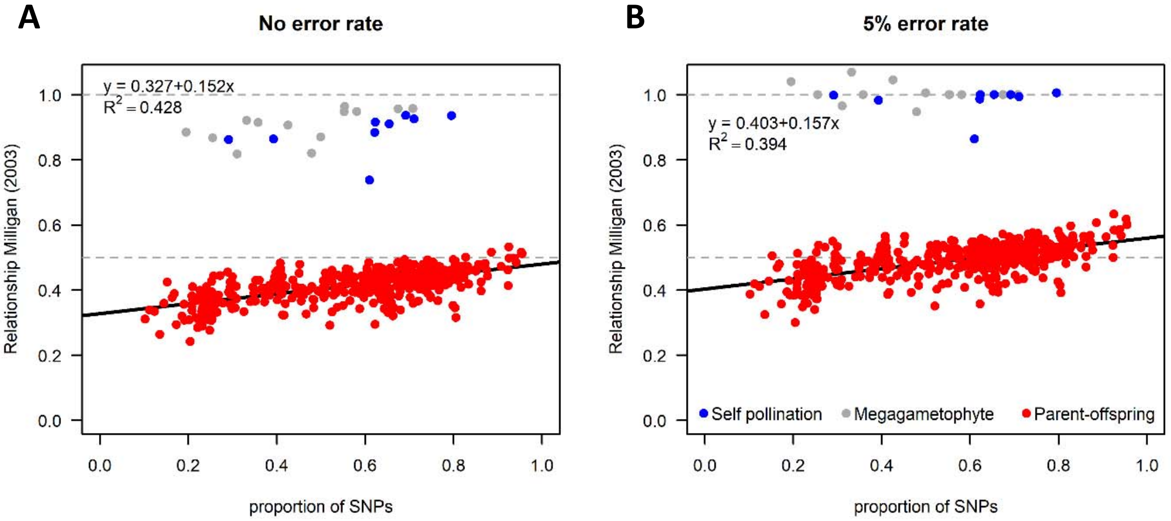

Figure S7. Milligan (2013) maximum likelihood relationship estimates for parent offspring pairs. In pane **A**, no genotyping error/genotype call error is assumed while pane **B** assumes that about 5% of the calls are erroneous.

Parentage and relatedness reconstruction in *Pinus sylvestris* using genotyping by sequencing.

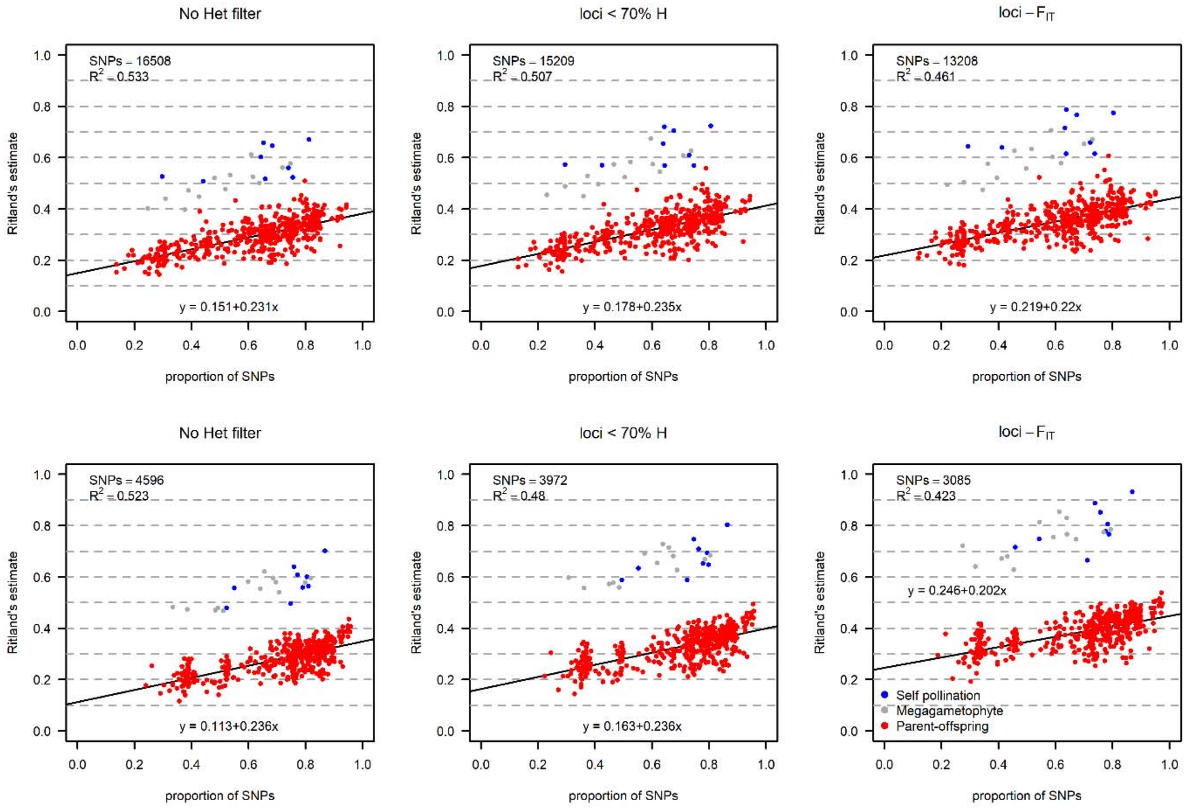

Fig. S8. Scatterplots of all pairwise comparisons of orchard genotypes (potential parents) and their offspring (seedlings). Filtering of SNPs follows the Material and Methods section except for MAF, which is 5% and the heterozygosity filtering which is different for each scatterplot above, leftmost no filtering of heterozygosity level of a locus, middle plot is 70% heterozygotes or less while right most plot shows the result from filtering negative F_{IT}. Scatterplots show Ritland's estimate based on all SNPs available for each comparison (shared SNPs for each two sample comparison) given as a proportion of the total number of SNPs after filtering. Bottom row is more rigorous filtering only allowing for 20% missing and a MAF of 10%. Blue filled circles are those assumed to be the product of self-pollination, grey are haploid megagametophyte material and red, parent offspring.

Supporting information for Hall D, Zhao W, Wennström U, Andersson Gull B and Wang XR

Parentage and relatedness reconstruction in *Pinus sylvestris* using genotyping by sequencing.

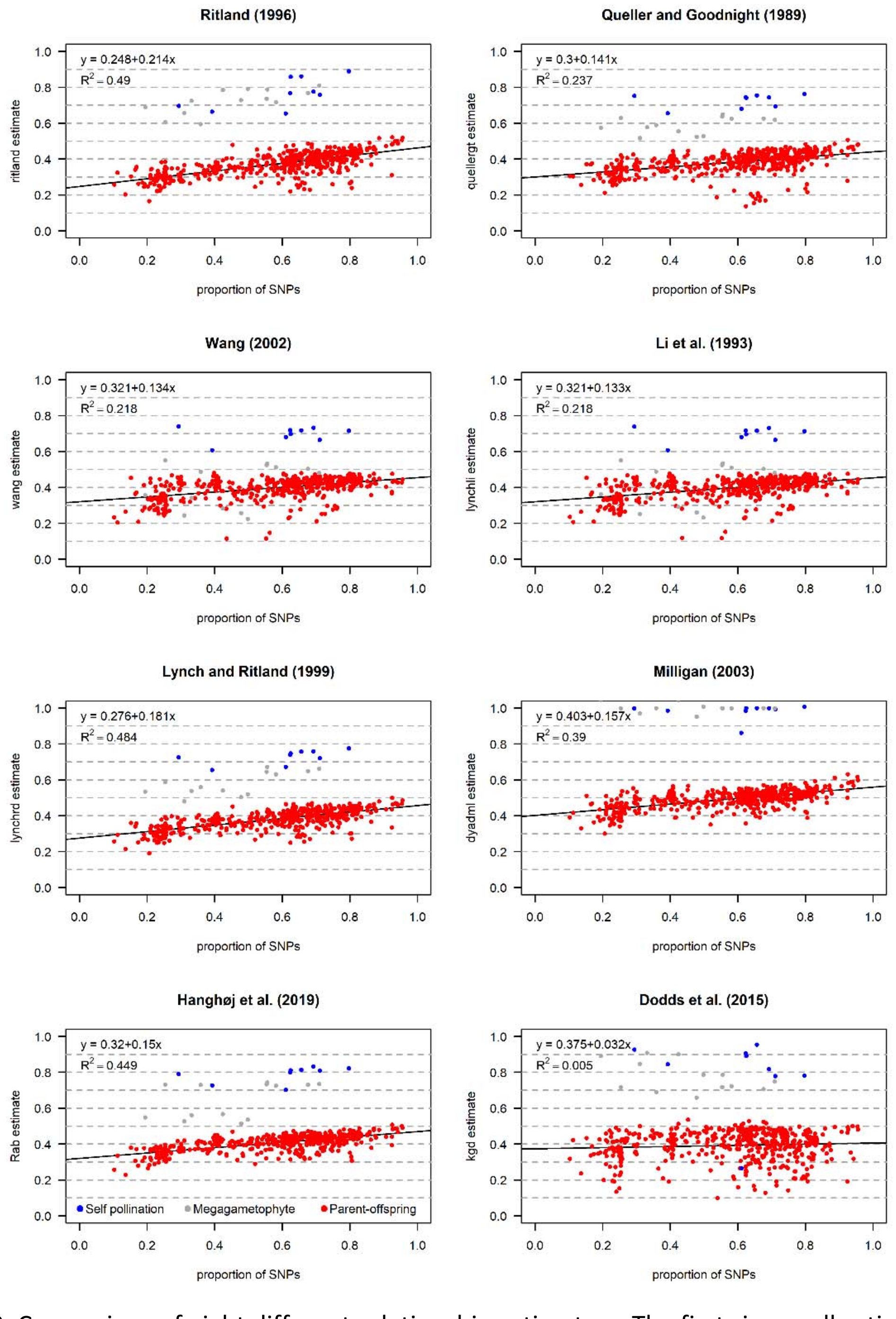

Fig. S9. Comparison of eight different relationship estimators. The first six are all estimated in the R-package 'related' (Pew *et al*, 2015; Wang, 2011) while the final two are from 'ngsRelate v2' (Hanghøj *et al*, 2019) which estimate the relationship between a and b through the Jacquard coefficients of IBD and 'KGD' (Dodds *et al*, 2015) respectively. Only *dyadml* from Milligan (2003) and the estimate from Hanghøj *et al* (2019) are maximum likelihood estimates where booth estimate the Jacquard coefficients of IBD (Jacquard, 1974) and then calculate relationship from those (dyadml = $2J_1 + J_3 + J_5 + J_7 + 0.5J_8$ and $R_{ab} = J_1 + J_7 + 0.75(J_3 + J_5) + 0.5J_8$ respectively.

Supporting information for Hall D, Zhao W, Wennström U, Andersson Gull B and Wang XR

Parentage and relatedness reconstruction in *Pinus sylvestris* using genotyping by sequencing.

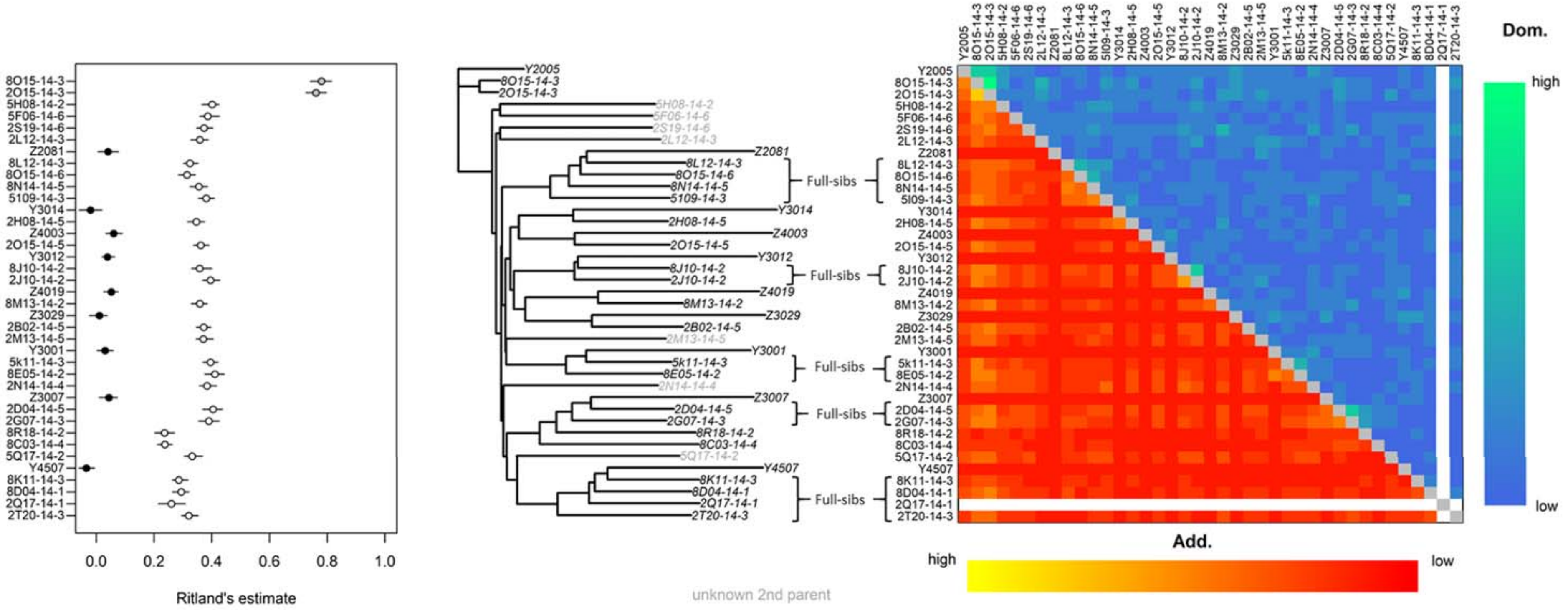

Figure S10. The extended family structure of parent Y2005 in the seed orchard crop. (**A**) Pairwise relationship estimates between Y2005 and its offspring (open circles) as well as other assigned parents (black filled circles) and seedlings categorized as a product of self-pollination (grey filled circles). (**B**) The relationship tree of Y2005's family structure and (**C**) the relative amount of shared additive genetic covariance and shared dominant genetic covariance between samples based on VanRaden (2008), seedling 2Q17-14-1 had too little coverage to be included in the G-matrix calculations.

Supporting information for Hall D, Zhao W, Wennström U, Andersson Gull B and Wang XR

Parentage and relatedness reconstruction in *Pinus sylvestris* using genotyping by sequencing.

References

Dodds KG, McEwan JC, Brauning R, Anderson RM, van Stijn TC, Kristjánsson T *et al* (2015). Construction of relatedness matrices using genotyping-by-sequencing data. *Bmc Genomics* **16**(1)**:** 1047.

Hanghøj K, Moltke I, Andersen PA, Manica A, Korneliussen TS (2019). Fast and accurate relatedness estimation from high-throughput sequencing data in the presence of inbreeding. *GigaScience* **8**(5).

Jacquard A (1974). *The genetic atructure of populations* Vol 5. Springer-Verlag: New York.

Li CC, Weeks DE, Chakravarti A (1993). Similarity of DNA fingerprints due to chance and relatedness. *Hum Hered* **43**(1)**:** 45-52.

Luu K, Bazin E, Blum MGB (2017). pcadapt: an R package to perform genome scans for selection based on principal component analysis. *Mol Ecol Resour* **17**(1)**:** 67-77.

Lynch M, Ritland K (1999). Estimation of pairwise relatedness with molecular markers. *Genetics* **152**(4)**:** 1753-1766.

Milligan BG (2003). Maximum-likelihood estimation of relatedness. *Genetics* **163**(3)**:** 1153-1167.

Pew J, Muir PH, Wang JL, Frasier TR (2015). related: an R package for analysing pairwise relatedness from codominant molecular markers. *Mol Ecol Resour* **15**(3)**:** 557-561.

Queller DC, Goodnight KF (1989). Estimating relatedness using genetic markers. *Evolution* **43**(2)**:** 258-275.

Ritland K (1996). Estimators for pairwise relatedness and individual inbreeding coefficients. *Genetical Research* **67**(2)**:** 175-185.

VanRaden PM (2008). Efficient methods to compute genomic predictions. *J Dairy Sci* **91**(11)**:** 4414-4423.

Wang J (2002). An estimator for pairwise relatedness using molecular markers. *Genetics* **160**(3)**:** 1203-1215.

Wang J (2011). COANCESTRY: a program for simulating, estimating and analysing relatedness and inbreeding coefficients. *Mol Ecol Resour* **11**(1)**:** 141-145.
